# Moral distress among midwives working in the labour ward of a hospital in Ghana

**DOI:** 10.18332/ejm/120117

**Published:** 2020-06-01

**Authors:** Bright Addo, Hannah A. Amoah, Sophia A. Eshun, Hannah N. Ocran

**Affiliations:** 1 Department of Sociology and Social Work, Kwame Nkrumah University of Science and Technology, Kumasi, Ghana; 2 Department of Nursing and Midwifery, University for Development Studies, Tamale, Ghana

**Keywords:** midwifery, Ghana, interpretative phenomenological analysis, moral distress, psycho-emotional wellbeing

## Abstract

**INTRODUCTION:**

The existing literature on moral distress is replete with studies conducted among nurses. That on midwives is however lacking. The aim of this study was to explore and understand moral distress from the perspective of and as experienced by midwives.

**METHODS:**

This study involved an exploratory and qualitative case study design and adopted the interpretative phenomenological approach to collect data from 8 Ghanaian female midwives working in the labour ward of a public Hospital in Sunyani, Ghana, using individual in-depth interviews. The data obtained through audio-taped recordings were transcribed verbatim and analysed, adopting Colaizzi’s qualitative analysis procedure.

**RESULTS:**

Three major themes were generated: perceived knowledge about moral distress, perceived causes of moral distress, and perceived effects of moral distress. The midwives perceived moral distress as stress arising from situations where they knew the right thing to be done or how to save a life but due to reasons such as resource constraints and disrespect from their colleagues and superiors, they were unable to do so. Moral distress often impacted negatively on midwives work performance and psycho-emotional wellbeing. Effects that manifested in the form of problems such as stomach sickness, sleepless nights, frustration, and anger, were mentioned.

**CONCLUSIONS:**

Efforts aimed at providing adequate logistics and resources, as well as improving communication, healthy interactions and interpersonal relationships among midwives and their colleagues should be intensified.

## INTRODUCTION

Defined as occurring *‘when one knows the right thing to do, but institutional constraints make it nearly impossible to pursue the right course of action’^1^*, moral distress continues to be one of the important issues in the field of clinical ethics that can have different effects not only on the performance of medical professionals such as physicians, nurses, midwives, and other healthcare providers, but also on patients and healthcare systems^2^.

A plethora of studies on moral distress, especially those conducted among nurses^1,3–5^ exist, and the knowledge buildup on the concept as applied to other medical fields, continues to grow. The growth in publications may be as a result of two key outcomes: 1) it may simply reflect the spread of an idea or a trend in academic interest, and 2) it may reflect an increase in the frequency, intensity, or extent of distress experienced among healthcare providers or professionals^1^.

Empirical studies on moral distress among midwives are seriously lacking. Indeed, a systematic review that investigated moral distress and its related factors revealed that most studies (72%) focused on nurses^2^, but recently, it has also been studied among physicians^6^, medical students^7^, and midwives^2,8^. The existing studies on moral distress among midwives have mostly adopted the quantitative approach. For example, the study of Zolala et al.^2^, which directly looked at moral distress among midwives, only estimated the severity and frequency of moral distress among midwives. Another study conducted by Oelhafen and Cignacco^8^, which assessed the burden associated with ethical issues, moral competences, and negative consequences of moral distress using a latent variable approach, was also purely quantitative. The quantitative approach adopted by these studies and others is short of the depth of information that would have been generated if these studies were qualitative.

In Ghana, research on moral distress among midwives and nurses is yet to be well demonstrated. The midwifery workforce in Ghana, like in other parts of the world, encounters morally distressing situations that affect the quality of care they provide to patients/clients, which need to be explored in-depth. This study, therefore, sought to explore and understand moral distress from the perspective of and as experienced by midwives working in the labour ward of a public hospital in Ghana.

## METHODS

### Study design

The study used an exploratory and qualitative case study design and the interpretative phenomenological approach to explore and understand what moral distress was and how it impacted midwives’ work output and well-being. The study was conducted at the labour ward of a public hospital in Sunyani, Ghana.

### Selection and description of study participants

Using the purposive sampling technique, 8 out of the 12 registered midwives working the three regular shifts (morning, afternoon, and night) in the hospital were selected. Selection of the 8 study participants was informed by Creswell^9^ recommendation of choosing between 5–25 participants for phenomenological studies, and the goal of attaining saturation by interviewing the eighth participant. The study participants’ ages ranged from less than 35 years to 45 or more years. Their years of working experience as midwives also ranged from less than 5 years to 15 or more years. They belonged to the Christian and Islamic faith, were either married or single or widowed, and occupied from staff midwife to principal midwifery officer ranks.

### Data collection, management, and analysis

Data for the study were obtained through the conduct of individual in-depth interviews. An interview guide that consisted of open-ended questions was developed and used. Informed consent was sought from study participants before interviews were carried out. All the participants voluntarily consented to participate in the study. Ethical issues relating to confidentiality and anonymity of participants were ensured. A voice recorder was used and notes taken during the interviews. The audio-taped (voice) recordings obtained after the interview sessions were transcribed verbatim. The accuracy of the transcripts was checked by reading them over and listening to the audio recordings for a second time. Analysis of the data collected was guided by the first five steps (Table 1) of Colaizzi’s distinctive seven-step process^10^.

**Table 1:** Steps in analysing data collected using Colaizzi’s descriptive phenomenological method

*Step*	*Description*
1. Familiarisation	All the transcripts were read through about five times to gain familiarity with the content.
2. Identifying significant statements	Statements in the accounts that were of direct relevance to the phenomenon under study (moral distress) were identified.
3. Formulating meanings	Meanings relevant to moral distress that emerged from a careful consideration of the significant statements were identified.
4. Clustering themes	Identified meanings were clustered into themes that were common across all accounts.
5. Developing an exhaustive description	Providing a full and inclusive description of the phenomenon (moral distress) which incorporated all the themes produced at the clustering step.

## RESULTS

The analysis generated three main themes: perceived knowledge about moral distress, perceived causes of moral distress, and perceived effect of moral distress.

### Perceived knowledge about moral distress

The midwives perceived moral distress to be stress arising from situations where they knew the right way something had to be done or how to save a life but due to reasons such as resource constraints and disrespect from their colleagues and superiors they were not able to do so. Moral distress was also viewed as a situation where patients either did not heed their instructions or patients sought further opinions/clarifications from other midwives, which created the impression that the midwife who provided the initial instruction or response to the patient’s question was incompetent.

*‘Moral distress occurs when a midwife knows the right way to save a life but there are no resources to help you.’* (MO)

*‘It occurs when there is a lack of respect from colleagues and superiors. When a junior colleague does not follow instructions that you give her and complications arise, you the senior are blamed.’* (PMO)

*‘Moral distress, well let's say a patient asks you something, and you tell her to do it this way and you see this patient again asking your colleague the same question…., it is very deep hurt. It's like the patient doesn't trust you but we all went to school and learnt the same thing.’* (SM1)

### Perceived causes of moral distress

The midwives were mostly of the view that lack of logistics, which was a major cause of moral distress, created a situation where the patients receiving care outnumbered the available resources to cater to them. This led to the midwives being unable to effectively and efficiently carry out their professional duties, which contributed to them becoming morally distressed. A dilemma of caregiving where a midwife’s recommendation for treatment was ignored by doctors, the unavailability or late response of doctors when midwives were faced with emergency situations, and disrespect from colleagues, were also mentioned as other perceived causes.

*‘It is caused by a lack of logistics and when the patients are more than midwives. For instance, I happen to be the only midwife on duty with one assistant managing the labour ward with about six patients in the active phase of labour. I became so tired and unable to monitor the patients while using pantograph, which resulted in the complication of one patient.’* (SM2)

*‘When I need assistance from a doctor, but I don't get, when complication set in, I am the one to blame.’* (MO2)

*‘I had to suture an episiotomy under a poor lighting system, which was a challenge to me because I did the suturing in a haphazard manner, I felt that what I did was unprofessional and might affect the woman.’* (SMO)

### Perceived effect of moral distress

The midwives reported the effects of moral distress that included low work output and incompleteness of care, anger, and frustrations, which resulted in shouting at patients, unprofessional conduct, and poor working relationships with their colleagues. Work fatigue, resulting in exhaustion that impacted midwives' ability to care for their home and the development of sleeping problems, was also mentioned.

‘Moral distress leads to low work output and sometimes I feel demoralized when I am in the ward and something bad happens to a patient or a baby, it spoils my day making me not working to my satisfaction.’ (MO3)

*‘In such situations, I feel very bad, and always think about the situation, I even ask myself if am in the right profession, I cannot give my family the needed attention, develop eating and sleeping problems sometimes. I have stomach problems, and sometimes feel like leaving the profession.’* (PMO)

## DISCUSSION

Consistent with the findings of others such as Burston and Tuckett^11^ and Maluwa et al.^3^, we found that moral distress is a phenomenon that is prevalent in the midwifery profession. Some of the midwives explained moral distress as a situation where a midwife may not be able to fully function or work well. Others also explained it as psycho-emotional disturbances or hurt feelings, which result from disrespect and confrontations with work colleagues, clients/patients or other health workers. Put together, these explanations corroborate the definition and explanations given by authors such as Jameton^1^ who described moral distress as feelings that are painful and which creates a psychological imbalance or disequilibrium often resulting from situations where one finds themselves in a position to do right instead of wrong^1^.

We identified the lack of logistics and inadequate human resources to be the significant perceived cause of moral distress in the midwifery practice. The inadequate/lack of logistics and human resources pose a significant challenge to the delivery of quality care to clients. This situation sometimes leads to improvisation in the delivery of care, which could be risky in critical situations. This finding is similar to that of Maluwa et al.^3^ who also found that midwives working in poorly resourced countries, like Malawi, struggle with inadequate supplies of drugs, safe water, medical equipment, and other essentials supplies. This results in the inability of midwives in these countries to provide the quality of care patients/clients deserve, which often leads to frustration and demoralization^3^. The experiences shared by the study participants also revealed that moral distress often impacted negatively on their wellbeing and work output. Effects that manifested in problems such as stomach problems, nightmares, loss of appetite, frustration, and anger, low work output, demoralization and dissatisfaction are in line with the extant literature^4,11,12^.

### Strengths and limitations

The findings of this study cannot, to a large extent, be generalized to the entire Ghanaian midwives’ community. Beyond generalization, however, this study is the first empirical investigation to adopt a qualitative approach to offering an in-depth understanding of moral distress as experienced by midwives. The results are therefore encouraging as they serve as a base for the advancement of knowledge in this area of research.

## CONCLUSIONS

Midwives play a critical role in achieving the SDG 3 targets 3.1 and 3.2, which aim at achieving a maternal mortality ratio of less than 70 per 100000 live births and a neonatal mortality of at least 12 per 1000 live births by the close of 2030^13,14^, and, as such, matters that affect the effective and efficient delivery of their services must not be taken lightly. The results of our study prove that like nurses, midwives also do experience moral distress. Drawing from the findings of the study on the causes of moral distress, we suggest that the government of Ghana, through the Ministry of Health ensures that logistics and resources needed by midwives to effectively carry out their duties are provided for. Efforts aimed at improving communication, healthy interactions and interpersonal relationships among midwives and their colleagues, which could be achieved through the conduct of periodic in-service training, are also suggested.
